# Service preferences among postpartum women (non-)affected by different types of intimate partner violence: insights from the cross-sectional study INVITE

**DOI:** 10.1186/s12889-024-20921-y

**Published:** 2025-01-02

**Authors:** Laura M. Hausmann, Lara Seefeld, Amera Mojahed, Judith T. Mack, Susan Garthus-Niegel, Julia Schellong

**Affiliations:** 1 Institute and Policlinic of Occupational and Social Medicine, Faculty of Medicine, TUD Dresden University of Technology, Dresden, Germany; 2 Department of Psychotherapy and Psychosomatic Medicine, Faculty of Medicine and University Hospital Carl Gustav Carus, TUD Dresden University of Technology, Dresden, Germany; 3 Institute for Systems Medicine (ISM) and Faculty of Human Medicine, MSH Medical School Hamburg, Hamburg, Germany; 4 Department of Childhood and Families, Norwegian Institute of Public Health, Oslo, Norway; 5 Department of Psychology, Faculty of Social Sciences, Vytautas Magnus University, Kaunas, Lithuania

**Keywords:** Intimate partner violence, Postpartum period, INVITE study, Help seeking, Service preferences, Service provision, Cross-sectional study

## Abstract

**Background:**

Women in the postpartum period are at greater risk of intimate partner violence (IPV), which may cause physical, sexual, or psychological harm and have a long-lasting negative impact on mother and child. Seeking help in case of IPV in the postpartum period can be difficult.

**Objective:**

The purpose of this study was to examine service preferences among postpartum women in Germany (non-)affected by IPV.

**Methods:**

In the cross-sectional study INVITE, postpartum mothers (*n* = 3,509) were interviewed via telephone. Using the WHO-Violence Against Women Instrument (WHO-VAWI), women were divided into groups: non-affected women and women affected by psychological, physical, and/or sexual IPV.

Using analyses of variance, group differences regarding preferred services and modes of service provision were assessed. Examined service domains were psychosocial services (e.g., women´s shelter or self-help groups), medical services (e.g., gynecologist or emergency room), and midwives. Modes of service provision included direct communication (e.g., in person or video conference) and indirect communication (e.g., chat or e-mail).

**Results:**

People from the women's social environment (e.g., family, friends) and specialized IPV services, such as women's shelters, were the most preferred support. Regarding service categories, women who experienced any type of IPV rated all three service domains less likely to be used than non-affected women. Most preferred provision mode was “in person”. Women affected by physical and/or sexual IPV rated direct modes more negatively than non-affected women. However, there were no differences between (non-)affected women regarding indirect modes, such as e-mails or apps.

**Discussion:**

The present results indicate that services were rated less likely to be used by postpartum women affected by IPV. Potential barriers which lead to these ratings need to be investigated. Efforts should be made to increase awareness of IPV and the beneficial effects of support.

## Background

### Intimate partner violence and help seeking

Intimate partner violence (IPV) constitutes the most common form of violence against women [1, 2] and is defined as any behavior by a current or former intimate partner that causes physical, sexual, or psychological harm, including acts of physical aggression, sexual coercion, psychological abuse, and controlling behaviors [3]. According to the violence against women EU-wide survey, 43% of women in the EU who had a (current or previous) partner reported experiences of psychological IPV and 22% of women reported physical and/ or sexual IPV since the age of 15 [1]. In Germany, lifetime prevalence of IPV was 57.6% of women (14 years and older). Out of the different subtypes, psychological IPV was most prevalent with 53.6%, while 15.2% of women reported physical IPV and 18.6% reported sexual IPV, and all forms regularly coincided [4]. In the first year postpartum, the prevalence of IPV worldwide ranges from 2% in Sweden to 58% in Iran [5].

**Fig. 1:**
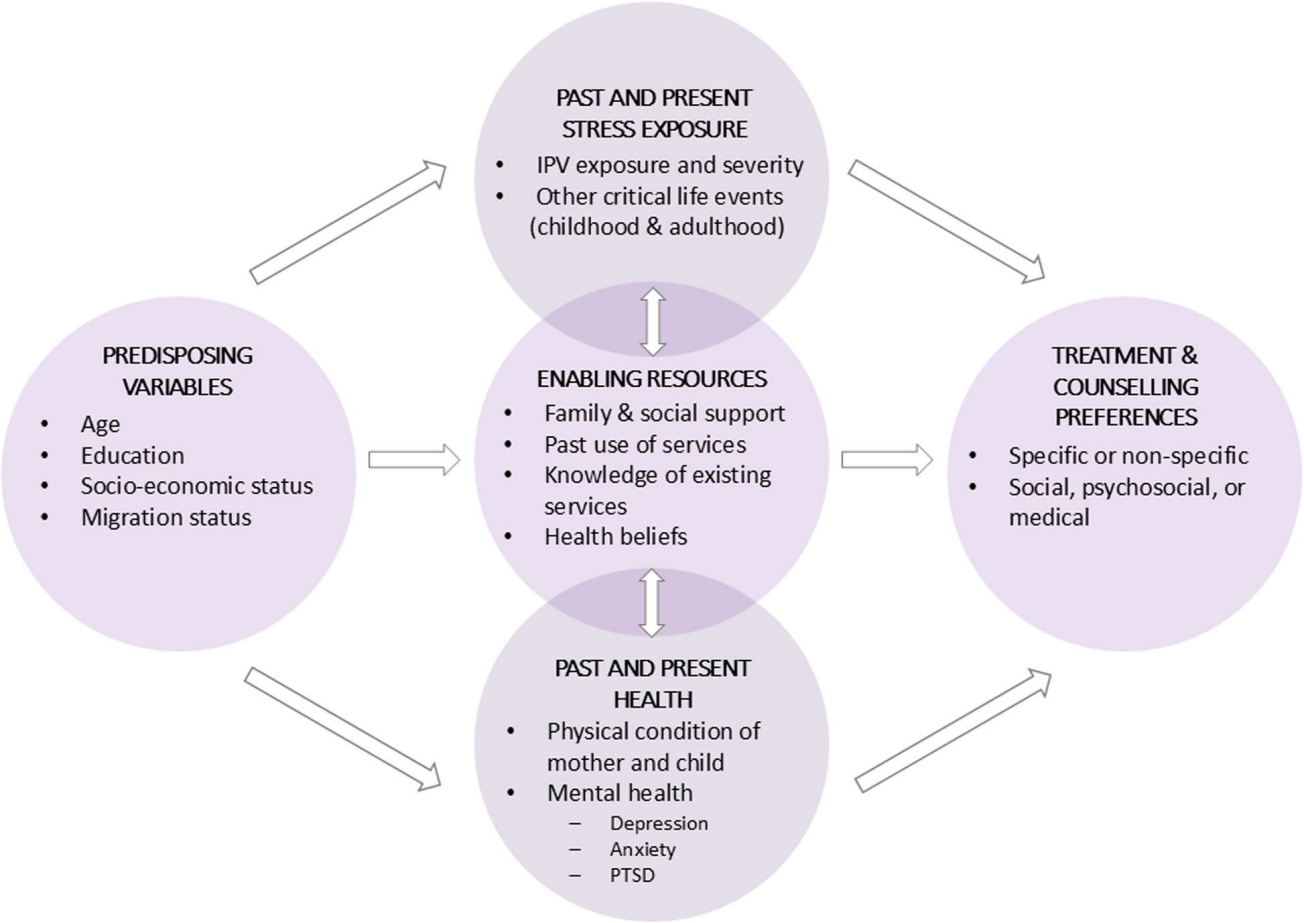
Predictors of Help Seeking Preferences: Theoretical Framework of the INVITE Study. *Note.* Theoretical Framework of the INVITE study, adapted version of the Andersen´s Behavioral Model of Access to Health Care [19] and a model from Liang et al. [20]. From “Preferences and barriers to counseling for and treatment of intimate partner violence, depression, anxiety, and posttraumatic stress disorder among postpartum women: study protocol of the cross-sectional study INVITE.” by Seefeld, L., Mojahed, A., Thiel, F., Schellong, J., and Garthus-Niegel, S., 2022, Frontiers in psychiatry, 13 (https://doi.org/10.3389/fpsyt.2022.836350). CC BY 4.0

In addition to often facing substantial physical and mental health challenges during the postpartum period [6], one German study suggests that the birth of a child can also be the catalyst for IPV [7]. Women who had experienced IPV reported worse health across a number of health domains in the immediate period after childbirth [8–11] and across the lifespan [12, 13], highlighting the importance of providing support to postpartum women experiencing IPV. Although any kind of support can improve the recovery and safety of women experiencing IPV [14–17], in Germany, more than two thirds of women suffering from physical or sexual IPV do not seek formal help [7]. Even in the case of informal help (e.g., family and friends), the proportion of women who seek support is alarmingly low [3, 18]. Consequently, approximately four out of ten affected German women do not tell anyone about the violence [7]. Based on Andersen´s Behavioral Model of Access to Health Care [19] and a model from Liang et al. [20] the process of help seeking starts with problem recognition being influenced by social and cognitive factors, leading to a decision to act and, at last, selecting a source of help. The cross-sectional study INVITE (INtimate partner VIolence Treatment prEferences; [21]), on which the current study is based, integrated these two existing models of help-seeking into its theoretical framework and adjusted these to relevant aspects of motherhood (see Fig. 1). This seems to be important because especially postpartum women may face specific barriers that make it difficult to seek help, such as fear of being separated from their children, e.g., by losing custody [18, 22–25], or the focus on preserving family unity [25–27]. At the same time, studies have shown that becoming a mother can motivate affected women to seek help [28–31]. Therefore, it is particularly important to investigate women’s preferences for services in the postpartum period to best support them in seeking help and to prevent intergenerational impacts of IPV on families [32].

### Treatment and counseling services for IPV

Previous research has shown that women’s decision to seek help is facilitated by offering a variety of services that meet their needs [33]. Services can be classified into domains depending on the service provider, setting, and type of provided help. There is informal support (e.g., family and friends) and formal support services, which contain counseling services (e.g., family counseling centers), specific domestic violence services (e.g., women’s shelters), criminal justice services, or medical services (e.g., emergency room, general practitioner) [34]. During the perinatal period, women have frequent interactions with healthcare services and previous research suggests that this may offer an opportunity to help women affected by IPV [32, 35]. However, services often used by women in the perinatal period, such as gynecologists and midwives, have so far not been investigated as a support service for women experiencing IPV. Additionally, most previous studies have investigated affected women’s satisfaction with the services received and have neglected affected women who have not sought help yet [7, 36, 37]. Ensuring accessibility and tailored services is essential for these women. They may be unaware of the support available, so exploring their preferences and offering appropriate services may enable them to seek help when they need it. Taking these preferences into account promotes prevention, early identification, accessibility, and empowerment of women. Therefore, it is particularly important to include women who have not yet sought or received help in studies investigating service preferences.

Research so far suggests that women choose different services according to their needs [20, 38]. For instance, those who need emotional support tend to seek informal help, while those who need immediate medical assistance are more likely to seek formal help [39–43]. Further, prior studies showed that the type of violence influenced the type of service approached [44, 43]. Associations were found between psychological violence and informal help [39–44]. While some studies revealed that experiences of physical IPV led more frequently to seeking formal [7], another study found that participants were more likely to seek informal help for physical IPV [34]. Experiences of sexual IPV were associated with increased help-seeking in the medical and psychosocial service domains and decreased informal help-seeking [41, 45]. Combined physical and sexual IPV was associated with increased help-seeking regarding psychosocial services, including community and IPV-specific services [45, 46]. These heterogeneous findings may be due to the fact that different studies assessed different service domains and not always all types and combinations of IPV. The extent to which different types of IPV may impact the preferences for specific service domains and settings in which services may be provided needs to be investigated in more detail.

### Mode of service provision

Services can be provided directly (e.g., in person or via video conference) or indirectly (e.g., via chat, e-mail or mobile phone app), with the latter meaning, that communication can be delayed in time. The number of service provision modes is constantly growing [47]. Prior studies indicated that some women experience indirect service provision as more accessible, while others prefer direct care [48]. Women affected by IPV who preferred indirect modes reported that seeking help this way was easier than in person because of anonymity and better accessibility at any time [49]. Indirect modes were perceived as a safe and confidential tool for initiating discussions about IPV with health professionals, assisting women in enhancing their safety and exploring help-seeking options [50]. This seems to be especially beneficial for women with social anxiety, when transportation is an issue [51], or in case of women whose partners control their physical whereabouts [52]. However, there are still challenges with indirect modes of service provision. There is not always access to a stable internet connection or an electronic device [48, 53] and some clients struggle with security risks (e.g., device security, children or abusers overhearing conversations, etc.) or technological skills [51]. In general, there is little research on preferences for different modes of service provision for non-affected and affected women in the postpartum period and in case of different types of IPV.

### Aims of this study

The aim of this study was to provide new insights into the preferences for counseling and treatment services along with the mode of service provision among postpartum women in Germany (non-)affected by IPV. The study extends previous research by examining a wide range of services for women in the postpartum period, specialized in IPV or more general services, in different settings, and with different modalities. It also fills a knowledge gap about preferences of women who have not yet received help, especially in the postpartum period and distributed by type of IPV. For this reason, we decided in favour of an exploratory data analysis.

Our objectives were to explore whether and how women with experiences of psychological, physical, and/or sexual IPV compared to women without experiences of IPV differ from each other in their ratings of preferences for counseling and treatment services (in total and in specific service domains) and for modes of service provision (in total and in specific provision domains).

## Methods

### Design

This study was based on data from the cross-sectional study INVITE, which examines the preferences for and barriers to counseling and treatment services of women in the postpartum period [21]. The aim was to investigate women's (postpartum) health and other factors that support access to appropriate services, especially in cases of mental health problems or exposure to IPV. For this purpose, women were contacted for a standardized telephone interview lasting approximately one hour three to four months postpartum. All participants provided written informed consent to participate.

### Sample

Recruitment for the study took place from November 2020 to October 2023 at various maternity hospitals and freestanding birth centers in the Dresden area, Germany – specifically at birth information events, home visits for new parents by the youth welfare office, midwife (antenatal) appointments, and on the maternity ward. Women who were younger than 16 years or who did not have sufficient language skills in German or English to take part in the study were excluded. Monetary compensation for study participation was 20 €.

**Fig. 2:**
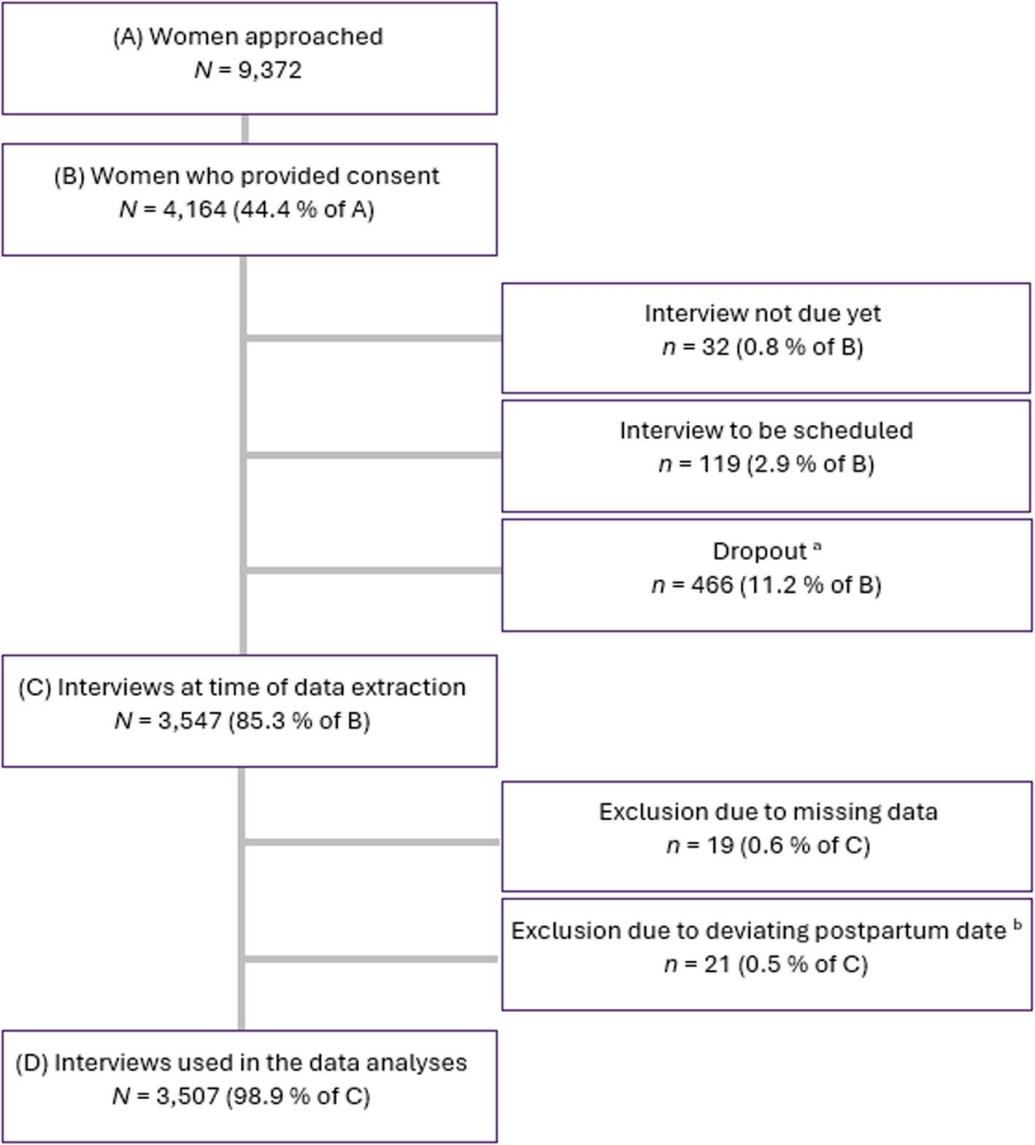
Study Population and Retention Rate. *Note. *The final sample size was based on recruitment between November 2020 and April 2023. ^a^ Due to consent being withdrawn or because the woman could not be reached. ^b^ Women less than six weeks or more than six months postpartum at the time of the interview

The current study is based on Version 3 of the quality-assured data files of the INVITE study (data extraction on 21.04.2023). At the time of data extraction, 4,164 of the 9,372 women approached gave consent to participate in the study, and 3,547 completed the interview. To obtain comparable results, women less than six weeks or more than six months postpartum at the time of the interview were excluded (*n* = 21). Additionally, *n* = 19 women were excluded due to missing data in all variables of interest. The retention rate of the study population is presented in Fig. 2.

### Measures

#### Independent variables

Women were divided into five groups: non-affected women, women affected by psychological IPV, women affected by physical IPV, women affected by sexual IPV, and women affected by both physical and sexual IPV. Previous studies have shown that there is a large overlap between psychological IPV and physical and/or sexual IPV [1, 4]. Therefore, women affected by psychological and physical as well as women affected by both psychological and sexual IPV were included in the physical and/or sexual IPV groups. The independent variables were assessed using the WHO-Violence Against Women Instrument (WHO-VAWI) [54] with behavior-specific items related to psychological (four items), physical (six items), and sexual IPV (three items). Respondents were categorized as being exposed to IPV if they answered any question affirmatively [54]. For each question, respondents were asked whether they had experienced the specific act during the past year and/or earlier in life. The instrument was translated and validated into German by the INVITE research team, following suggestions from the WHO [55]. In this sample, the internal consistency of the total score was good (Cronbach's *α* = 0.82). The internal consistency of subscales was acceptable for psychological IPV (McDonald´s *ω* = 0.69) and good for physical IPV (*ω* = 0.82) and sexual IPV (*ω* = 0.73).

#### Dependent variables

##### Counseling and treatment service preferences

The first main dependent variable was the total score of preferences for counseling and treatment services assessed with a self-generated questionnaire [56]. These services were of varying specializations within the German support system and ranged from very specific options tailored to IPV, such as women's shelters, to more general services for all women, such as general practitioners [21]. Women were asked to indicate on a four-point response scale (“not at all” to “definitely”) how likely it is that they would pick a specific service based on their preferences if they are or were affected by any type of IPV. Women who were not affected were asked to imagine the respective situation and answer the questions as if they were affected (i.e., hypothetically). The questionnaire consists of 19 items, which form the total score ranging between 19 and 76. The total score is used to determine how likely women generally are to seek help from any kind of service. Alongside this composite variable, subscales of services were computed and considered as distinct dependent variables. Therefore, an exploratory Principal Axes Factor Analysis (PFA) with varimax rotation was conducted to determine the factor structure of these dependent variables (see Appendix 1). Examination of Kaiser’s criteria and Bartlett’s test yielded justification for retaining three factors with eigenvalues exceeding 1, which accounted for 41.27% of the total variance. Three items (“family member, friend, or colleague”, “woman in the same situation”, and “religious institutions”) were excluded due to a factor loading below 0.30. The following subscales were derived: Psychosocial services (e.g., women´s shelter or self-help groups), medical services (e.g., gynecologist or emergency room), and midwives (midwives and family midwives). Internal consistency was good (*α* = 0.83) for the service preferences questionnaire, as well as for the subscales psychosocial (*ω* = 0.80) and medical services (*ω* = 0.79). Interitem correlation for the subscale midwives was high (0.64). Finally, participants were asked if they would contact the police or any legal service provider if they would experience IPV. Here, the answer options were “yes”, “no”, and “I don´t know”.

##### Mode of service provision preferences

The second main dependent variable was the total score of mode of service provision preferences for IPV assessed with a self-generated questionnaire [21]. The questionnaire consisted of seven items, and participants could answer on a four-point response scale (“not at all” to “definitely”), with a minimum total score of seven and a maximum total score of 28, whether the different options would meet their needs when using IPV services. A high total score indicates that women would seek help across all modes in case of IPV. Subscales for different modes of service provision were calculated with an exploratory PFA with varimax rotation (see Appendix 2) and considered as separate dependent variables to investigate whether there are differences in preferences for certain provision modes. Two factors explaining a total of 44.77% of the variance were revealed: Direct modes (i.e. in person, telephone call, and video conference) and indirect modes (i.e. app or online platform, chat or e-mail). In comparison to the direct modes, the indirect modes can be time-delayed and are less identifiable. Internal consistency was acceptable (*α* = 0.72) for the mode of service provision preferences questionnaire and good for the subscale direct modes (*ω* = 0.70) and the subscale indirect modes of service provision (*ω* = 0.70). In accordance with the assessment of counseling and treatment service preferences, participants not currently affected by IPV were asked to imagine that they were suffering from IPV.

#### Covariates

Based on their importance in the literature on postpartum help-seeking behavior [34, 57–59], the following variables were included as potential covariates: duration of residence in Germany, categorized as “born in Germany” and number of years since migration to Germany (< 5 years, 5–10 years, and > 10 years), net monthly household income (< 1,250 €, 1,250 €–2,249 €, 2,250 €–2,999 €, 3,000 €–3,999 €, 4,000 €–4,999 € and > 5,000 €), and number of children in total. Additionally, time of occurrence of IPV (experiences in lifetime versus within the last 12 months) was included as a potential covariate.

### Data analysis

For the following data analyses, IBM SPSS Statistics (Version 28.0.0.0) was used. First, the data set and all relevant variables were checked for outliers and extreme values. Univariate outliers were identified by using boxplots displaying the interquartile range (IQR). Values three times greater than the IQR were considered extreme univariate outliers. Multivariate outliers were identified using the Mahalanobis distance, which was based on robust estimations of the mean and covariance matrix. Assumptions for all analyses were tested. The main sociodemographic characteristics, potential confounding variables, predictors, and outcome variables were examined descriptively. Missing values of less than 20% in psychometric scales were replaced by the woman’s mean value. If a woman completed less than 80% of the items of a given questionnaire, the score for that questionnaire was treated as missing. However, responses from the same participant on other questionnaires were included in the subsequent analyses. Spearman correlation analyses were performed to identify relevant covariates. Variables which correlated significantly with a main dependent variable (total score of counseling and treatment service or total score of mode of service provision preferences) were included in the multivariate analyses as covariates.

Two one-way analyses of covariance (ANCOVA) were performed to investigate the differences between the total scores of service preferences as well as the mode of service provision preferences between all groups including identified covariates. As there was heterogeneity of covariates across groups for number of children and maternal age, main effects and interaction statistics were reported based on Wilks' lambda test statistic, following the recommendation of Ateş et al. [60]. Additionally, analyses were performed with and without these heterogeneous covariates to control for this unfulfilled assumption. As no difference in significance level was found, covariates were maintained. In the case of identified outliers, main analyses were reported without outliers, and sensitivity analyses were performed to control for their influence. Significant differences in these sensitivity analyses were reported. The ANCOVA for service preferences was calculated with the interaction term “maternal age*IPV group” to address the unmet statistical requirements of homogeneity of regression slopes. To reveal the differences between the groups and their relation to the subscales of service preferences as well as the subscales of preferences in modes of service provision, two one-way multiple analyses of covariance (MANCOVA) and post hoc tests were applied. Due to multiple testing, Bonferroni correction was applied. As there was no normal distribution of residuals, all analyses were performed with bootstrapping using the bias-corrected and accelerated (BCa) method with 95% confidence intervals (CIs). Analyses were calculated with 1,000 iterations.

In ANCOVAs and MANCOVAs SPSS uses listwise deletion as the standard method to handle missing data. This means that SPSS removes entire cases from the analysis if there are any missing values in any of the variables involved in that analysis. Therefore, *n* varied slightly between analyses.

Data collection and management were facilitated by using “Research Electronic Data Capture” (REDCap), a secure, web-based application for data capture as part of research studies, hosted at the ‘Koordinierungszentrum für Klinische Studien’ at the Faculty of Medicine of the Technische Universität Dresden [61, 62].

## Results

### Descriptive statistics

Mothers were on average 32.95 years old (*SD* = 4.63). Most of them were born in Germany (91.5%), had a partner (97.8%), and attained more than ten years of education (73.9%). Detailed sociodemographic characteristics of the sample are presented in Table 1.

**Table 1:** Sociodemographic characteristics of the sample

	*M (SD)*	Range
**Maternal age**^**a**^	32.95 ± 4.63	16.78–53.96
**Age of child**^**b**^	13.23 ± 2.73	6.00–25.57
	*n* = *3,507*	%
**Duration of residence in Germany**^**c**^		
Born in Germany	3,210	91.5
<5 years	87	2.5
5–10 years	103	2.9
>10 years	109	3.1
**Partnership status**		
Partner	3,432	97.8
No partner	77	2.2
**Education**		
≤ 10 years	914	26.0
> 10 years	2,592	73.9*
**Net income** ^**d**^		
< 1,250 €	84	2.4
1,250 €–2,249 €	356	10.2
2,250 €–2,999 €	440	12.6
3,000 €–3,999 €	895	25.6
4,000 €–4,999 €	883	25.3
> 5,000 €	837	24.0*
**Number of children**		
1	1,843	52.6
2	1,275	36.4
3	298	8.5
4	67	1.9
≥ 5	22	0.6
**IPV groups**		
Without	1,813	51.9
Psychological	941	26.9
Physical	278	7.9
Sexual	297	8.5
Sexual and physical	172	4.9

*n* varied slightly due to missing values, *IPV* Intimate Partner Violence

^a^in years, ^b^in weeks, ^c^time since migration to Germany, ^d^per month and household

^*^The valid percentages do not add up to 100% due to rounding errors

### IPV prevalence

**Fig. 3:**
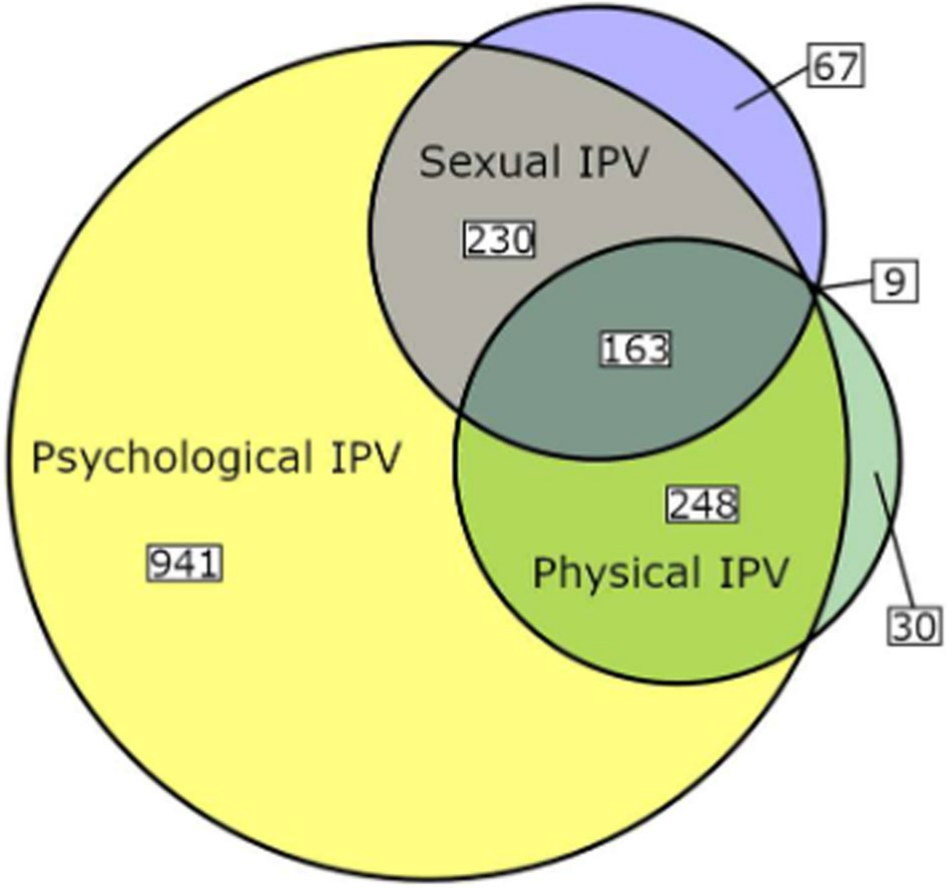
Overlap in Lifetime Prevalence for Different Types of IPV. *Note*. *IPV* Intimate Partner Violence. Proportions in relation to the total sample of affected women (*n *= 1,688)

In this sample, 48.2% of women disclosed instances of IPV. Specifically, 45.2% of women had encountered psychological IPV, with 13.5% experiencing it within the past 12 months. Additionally, 12.8% reported incidents of physical IPV, with 1.9% being affected in the last 12 months. Moreover, 13.3% of women were affected by sexual IPV, with 1.3% experiencing it within the past 12 months. As shown in Fig. 3, most women affected by sexual and/or physical IPV also reported experiences of psychological IPV, for which the highest lifetime prevalence was found.

### Preferences for counseling and treatment services and mode of service provision

Table 2 shows the descriptive statistics of preferences for all service and mode of service provision items across all groups. Among the various services, on average women tended to prefer the items “family member, friend, or colleague” and “woman in the same situation” the most and “religious institutions” the least. “In person” was the most popular modality of service provision, followed by “telephone call” and “video conference”. Women rated “apps or online platforms without guidance from an expert” as the least popular on average.

**Table 2:** Descriptive statistics of preferences for individual counseling and treatment services as well as for mode of service provision

Item	*n* = 3,507 *M (SD)*	*n*^*a*^ = 1,688 affected women *M (SD)*	*n*^*b*^ = 1,812 non-affected women *M (SD*)	Likeliness of picking a service:
**Counseling and treatment service preferences**				Definitely (4)
Family member, friend, or colleague	3.3 (0.8)	3.3 (0.8)	3.4 (0.7)	Rather yes (3)
Woman in the same situation	3.2 (0.7)	3.2 (0.7)	3.2 (0.7)	
Women's shelter	3.1 (0.8)	3.0 (0.8)	3.1 (0.8)	
Intervention center	3.0 (0.7)	3.0 (0.7)	3.1 (0.7)	
Emergency Room	3.0 (0.9)	2.9 (0.9)	3.0 (0.8)	
Help hotline for violence against women or sexual abuse	2.9 (0.8)	2.9 (0.7)	3.0 (0.8)	
Life and family counseling center	2.9 (0.7)	2.9 (0.8)	2.9 (0.7)	
Family midwife	2.9 (0.9)	2.9 (0.8)	2.9 (0.8)	
Counseling service for victims of crime	2.9 (0.8)	2.8 (0.8)	2.9 (0.7)	
Midwife	2.8 (0.9)	2.8 (0.9)	2.9 (0.9)	
Gynecologist	2.8 (0.9)	2.8 (0.9)	2.8 (0.9)	
Outpatient clinic for psychiatry or psychosomatic medicine	2.8 (0.8)	2.8 (0.9)	2.7 (0.8)	
General Practitioner	2.7 (0.9)	2.6 (0.9)	2.7 (0.9)	
Self-help group	2.6 (0.9)	2.6 (0.9)	2.7 (0.9)	
Psychosocial crisis service of the health department	2.5 (0.8)	2.5 (0.8)	2.5 (0.8)	Rather no (2)
Telephone counseling	2.5 (0.8)	2.5 (0.8)	2.5 (0.8)	
Social pedagogical family assistance	2.5 (0.8)	2.4 (0.8)	2.5 (0.8)	
Pediatrician	2.3 (1.0)	2.3 (1.0)	2.3 (0.9)	
Religious institutions	1.7 (0.8)	1.6 (0.8)	1.8 (0.9)	
				Not at all (1)
**Mode of service provision preferences**				Definitely (4)
In person	3.7 (0.5)	3.7 (0.6)	3.7 (0.5)	
Telephone call	2.7 (0.8)	2.7 (0.8)	2.7 (0.7)	Rather yes (3)
Video conference	2.6 (0.8)	2.6 (0.8)	2.7 (0.8))	
App or online platform with guidance from expert	1.9 (0.8)	1.9 (0.8)	2.0 (0.8)	Rather no (2)
Chat	1.8 (0.8)	1.9 (0.8)	1.8 (0.7)	
E-mail	1.8 (0.8)	1.8 (0.8)	1.8 (0.8)	
App or online platform without guidance from expert	1.5 (0.6)	1.5 (0.6)	1.5 (0.6)	Not at all (1)

*n *varied slightly due to missing values

In addition to these services, women were asked if they would contact the police or any other law enforcement agency. In this sample, 2,522 women (71.9%) affirmed that they would contact the police, while 728 women (20.7%) answered “don´t know”, and 259 women (7.4%) answered “no”.

### Correlation analysis

Spearman’s rank correlation was computed to assess the relationships between covariates and outcome variables. A higher score of counseling and treatment service preferences was statistically significantly associated with higher maternal age (*r* = 0.053, *p* = 0.001). A higher score of modes of service provision preferences was statistically significantly associated with a shorter duration of residence in Germany (*r* = −0.044, *p* = 0.001) and a lower number of children (*r* = −0.043*, p* = 0.05). Therefore, maternal age was included in the following analysis for service preferences (for the total score as well as for every service subscale). Likewise, duration of residence in Germany and number of children were included in the analysis for mode of service provision preferences. Detailed correlations can be found in Appendix 3.

### Group comparisons for counseling and treatment service preferences

After adjusting for maternal age and its interaction term due to unmet homogeneity requirement (maternal age*IPV groups), IPV groups differed significantly in their total scores for preferences for services [*F*(4,3489) = 3.161, *p* = 0.013, partial *η*^*2*^ = 0.004]. Bonferroni-corrected post hoc tests revealed a lower total score for counseling and treatment service preferences for women affected by any type of IPV compared to women without experiences of IPV, indicating that they preferred all services less than non-affected women. Furthermore, the total score for preferred services of women affected by physical IPV or women affected by sexual IPV was significantly lower than that of women affected by psychological IPV (see Table 3). Neither maternal age as a covariate [*F*(1, 3489) = 1.931, *p* = 0.165] nor its interaction term [*F*(1, 3489) = 2.741, *p* = 0.098] showed statistical significance.

**Table 3:** Group comparisons for counseling and treatment service preferences

IPV group (I)	IPV group (J)	Services				Psychosocial services				Medical services				Midwives			
		*M (I─J)*	*P*	*95 % BCa CI*		*M (I─J)*	*p*	*95 % BCa CI*		*M* *(I─J)*	*p*	*95 % BCa CI*		*M (I─J)*	*p*	*95 % BCa CI*	
				*LL*	*UL*			*LL*	*UL*			*LL*	*UL*			*LL*	*UL*
Without	Psychological	2.06*	.015	0.53	3.73	0.57**	.004	0.22	0.96	0.27**	.016	0.06	0.48	0.20**	.002	0.08	0.33
	Physical	4.42**	.007	1.51	7.94	1.44***	<.001	0.84	2.06	0.39*	.032	0.02	0.74	0.43***	<.001	0.23	0.63
	Sexual	5.32*	.023	1.10	10.37	1.35***	<.001	0.76	1.95	0.28	.092	-0.05	0.63	0.47***	<.001	0.26	0.66
	Sexual and physical	6.28*	.040	0.81	12.71	1.25**	.008	0.39	2.13	0.32	.170	-0.12	0.80	0.38***	<.001	0.14	0.61
Psychological	Without																
	Physical	2.37**	.008	0.79	4.19	0.87*	.011	0.23	1.56	0.12	.540	-0.28	0.48	0.22*	.039	0.02	0.43
	Sexual	3.26*	.046	0.33	6.68	0.77*	.014	0.12	1.44	0.01	.943	‑0.35	0.37	0.26*	.018	0.03	0.47
	Sexual and physical	4.22	.065	0.18	9.04	0.68	.148	-0.21	1.62	0.05	.830	-0.45	0.55	0.18	.160	-0.09	0.43
Physical	Without																
	Psychological																
	Sexual	0.90	.342	-0.95	2.99	-0.10	.808	-0.95	0.64	-0.10	.642	-0.58	0.35	0.04	.760	-0.24	0.29
	Sexual and physical	1.86	.238	-1.12	5.30	-0.19	.709	-1.28	0.82	-0.07	.836	-0.60	0.49	-0.05	.736	-0.34	0.22
Sexual	Without																
	Psychological																
	Physical																
	Sexual and physical	0.96	.321	-0.93	3.21	-0.10	.859	-1.04	0.95	0.04	.862	-0.46	0.59	-0.09	.570	-0.37	0.21
Sexual and Physical	Without																
	Psychological																
	Physical																
	Sexual																

*IPV* Intimate Partner Violence. Bootstrap results are based on 1,000 bootstrap samples. *BCa CI* bias-corrected and accelerated bootstrap interval, *LL* lower limit, *UL* upper limit. Calculations without outliers and with covariate: maternal age

*p < .05. **p < .01. ***p < .001

Considering group comparisons for the three subscales of service preferences, 15 multivariate outliers were identified and excluded from further calculations. After adjusting for maternal age, one-way MANCOVA with bootstrapping revealed differences between groups and the subscales [*F*(12, 9186.340) = 5.806, *p* < 0.001, partial *η*^*2*^ = 0.007, Wilk’s *Λ* = 0.980]. Moreover, maternal age as a covariate showed significance [*F*(3, 3472.000) = 12.029, *p* < 0.001, partial* η*^2^ = 0.010, Wilk’s* Λ* = 0.990].

Post hoc univariate ANOVAs were conducted for the three subscales and indicated statistically significant differences between IPV groups in every subscale, namely preferences for psychosocial services [*F*(4, 3474) = 11.105, *p* < 0.001, partial *η*^*2*^ = 0.013], medical services [*F*(4, 3474) = 2.665, *p* = 0.031, partial *η*^2^ = 0.003], and midwives [*F*(4, 3474) = 10.755, *p* < 0.001, partial *η*^2^ = 0.012].

Bonferroni-corrected post hoc analyses with bootstrapping revealed that psychosocial services were less preferred by women affected by any type of IPV compared to non-affected women. Furthermore, women affected by physical IPV and women affected by sexual IPV reported that they would be less likely to use psychosocial services than women affected by psychological IPV. Detailed information can be found in Table 3.

Medical services were rated significantly less likely to be used by women affected by psychological IPV and women affected by physical IPV than by non-affected women. In this subscale, higher maternal age was additionally associated with significantly higher ratings for medical service preferences [*F*(1, 3474) = 26.678, *p* < 0.001, partial *η*^*2*^ = 0.008].

As shown in Table 3, midwives were significantly less likely to be used by women affected by any type of IPV compared to non-affected women, and women affected by physical or sexual IPV rated midwives less likely to be used than women affected by psychological IPV. According to Cohen, the effect sizes were small for the subscales of psychosocial service preferences (partial *η*^*2*^ = 0.013) and preferences for midwives (partial *η*^*2*^ = 0.012) and very small for the subscale of medical service preferences (partial *η*^*2*^ = 0.003).

### Group comparisons for mode of service provision preferences

Considering mode of service provision preferences, one univariate extreme outlier was found and excluded from the calculations. IPV groups did not differ significantly in their preferences for modes of service provision in the total score [*F*(4,3491) = 1.290, *p* = 0.272, partial *η*^*2*^ = 0.001] after adjusting for number of children and duration of residence in Germany. Both covariates, however, showed statistical significance: a longer duration of residence in Germany [*F*(1, 3491) = 9.800, *p* = 0.002, partial *η*^*2*^ = 0.003] and a higher number of children [*F*(1, 3491) = 11.132, *p* = 0.001, partial *η*^*2*^ = 0.003] were associated with lower scores in preferences for mode of service provision.

Considering group comparisons for the two subscales of mode of service provision preferences, 24 outliers were found and excluded. Groups differed on the subscales as revealed with a one-way MANCOVA [*F*(8, 6932.00) = 2.569, *p* = 0.009, partial *η*^*2*^ = 0.003, Wilk’s *Λ* = 0.994)]. Duration of residence in Germany [*F*(2, 3466.000) = 22.588, *p* < 0.001, partial *η*^*2*^ = 0.013, Wilk’s *Λ* = 0.987] and number of children [*F*(2, 3466.000) = 7.526, *p* < 0.001, partial *η*^*2*^ = 0.004, Wilk’s *Λ* = 0.996)] were also statistically significant in this model.

Post hoc univariate ANOVAs showed a statistically significant difference between IPV groups for the subscale of direct modes of service provision [*F*(4, 3467) = 4.577,* p* = 0.001, partial *η*^*2*^ = 0.005] but not for the subscale of indirect modes of service provision [*F*(4, 3467) = 0.259, *p* = 0.904, partial *η*^*2*^ < 0.001]. The covariate number of children showed statistical significance for the subscale of direct modes of service provision [*F*(1, 3467) = 14.222,* p* < 0.001, partial *η*^*2*^ = 0.004] and the covariate duration of residence in Germany for the subscale of indirect modes of service provision [*F*(1, 3467) = 32.533,* p* < 0.001, partial *η*^*2*^ = 0.009].

Higher values of the covariates were associated with lower scores for preferences in the dependent variables. Bonferroni-corrected post hoc tests with bootstrapping for the subscale direct modes of service provision revealed that women affected by physical and/or sexual IPV rated direct modes less favorably than women without experiences of IPV (see Table 4). Likewise, women affected by physical IPV and/or sexual IPV rated direct modes less favorably than women affected by psychological IPV.

**Table 4:** Group comparisons for the subscale direct modes of service provision preferences

IPV group (I)	IPV group (J)	*M* (I─J)	*p*	95% *BCa CI*	
				*LL*	*UL*
Without	Psychological	0.00	.903	-0.04	0.04
	Physical	0.10**	.005	0.03	0.16
	Sexual	0.07*	.022	0.01	0.13
	Sexual and physical	0.10*	.017	0.02	0.19
Psychological	Without				
	Physical	0.09*	.010	0.02	0.17
	Sexual	0.07*	.032	0.00	0.13
	Sexual and physical	0.10*	.019	0.01	0.19
Physical	Without				
	Psychological				
	Sexual	-0.03	.474	-0.12	0.06
	Sexual and physical	0.01	.898	-0.10	0.12
Sexual	Without				
	Psychological				
	Physical				
	Sexual and physical	0.04	.494	-0.07	0.14
Sexual and Physical	Without				
	Psychological				
	Physical				
	Sexual				

*IPV* Intimate Partner Violence, Bootstrap results are based on 1,000 bootstrap samples, *BCa CI* bias-corrected and accelerated bootstrap interval, *LL* lower limit, *UL* upper limit. Calculations without outliers and with covariates: duration of residence in Germany, number of children

*p < .05. **p < .01

## Discussion

The aim of this study was to provide new insights into the preferences for counseling and treatment services for IPV and the preferred modes of service provision among women in the postpartum period. It was investigated whether and how postpartum women who had experienced different types of IPV and postpartum women who had not experienced IPV differ in their preferences.

Key findings were as follows: 1) Descriptive data revealed that people from the women's social environment and direct modes of service provision were the most preferred types of support among all women. 2) Counseling and treatment services overall were less likely to be used by women who experienced any type of IPV compared to non-affected women (hypothetically). 3) In the specific service domains, women affected by any type of IPV were less likely to use psychosocial services and seek help by midwives compared to non-affected women (hypothetically). Medical services were rated less likely to be used by women affected by psychological and/or physical IPV than by non-affected women (hypothetically). 4) There were no group differences in the preferences for using services via different modes in total and the indirect modes of service provision. 5) Direct modes were rated less likely to be used by women affected by physical and/or sexual IPV than by non-affected women (hypothetically).

### Counseling and treatment service preferences

Descriptive findings indicate that, among the array of services available, conversing with a "family member, friend, or colleague" emerged as the most preferred option on average across all women. A recent survey also showed that women affected by IPV were most likely to seek help from their family or friends compared to formal support services [63]. The recourse to informal support frequently marks the initial stride towards seeking help and exerts a notable influence on the determination to pursue formal help [7, 63–66]. Subsequently, women's shelters emerged as the preeminent service of choice among respondents. This preference may be attributed to the fact that the responsibility of women's shelters in cases of IPV is clearly evident, and the women may be able to anticipate the type of support they can expect from this service. In Germany, women’s shelter organizations form a national network and offer a wide range of services [67] Also thanks to a variety of public campaigns, women's shelters in Germany have become more widely known [68]. The emergency room was descriptively rated as the most preferred medical service among all respondents. This shows that women would be likely to seek help to treat current physical injuries but not necessarily with the intent to do something about the IPV itself. This is consistent with previous findings [63, 69, 70] and indicates the important role of well-trained professionals in medical settings to screen for IPV and provide appropriate support [71, 72].

Group comparisons revealed that women who have actually experienced IPV were less likely to use counseling and treatment services if needed than non-affected women would hypothetically do. This seems to be in line with the often reported low numbers of help seeking in case of IPV [3, 7, 33, 73]. Our findings indicate a trend wherein preferences, both across the total score and the subscales, exhibit a decline corresponding to the type of IPV: Women affected by physical and/or sexual IPV consistently assign the lowest ratings, followed by those experiencing psychological IPV, and subsequently by non-affected women. The decision to seek help is described as a process, which includes three stages: problem recognition, the decision to seek help, and last, the selection of a help provider [20, 63]. In the INVITE study, non-affected women were asked to hypothetically put themselves in the position of an affected woman. This implies that non-affected women could evaluate services without having experienced IPV and did not have to go through the first two stages of the help-seeking process [74]. Women who have not experienced IPV may struggle to empathize with the profound impact it can have. In addition to potentially being dependent on their abuser, low ratings in preferences for services may be caused by the psychological consequences of IPV, such as internal changes impacting their self-esteem, self-confidence, and self-efficacy as well as depression or feelings of stigma, shame and fear, which impede help-seeking [24, 69, 75, 76]. The majority of affected women in our sample reported experiences with IPV more than 12 months ago. The fact that these women nevertheless would be less likely to seek help may underline the seriousness of exposure to IPV and its long-lasting effects [77, 78]. Even if women affected by psychological IPV reported higher ratings than women affected by physical or sexual IPV, they were still significantly less likely to use counseling and treatment services than non-affected women. This is an important finding, because psychological IPV is often not recognized as abuse by the women themselves [76, 79, 80] and may support previous results about the seriousness of psychological IPV in particular [78, 81]. Furthermore, it is possible that women affected by IPV already had negative experiences with certain services and were therefore reluctant to use them again, because the first experience with psychosocial or medical service providers has a crucial impact on women’s future help-seeking decisions [18, 27, 63].

Finally, previous research outlined the opportunity of service provision by perinatal medical care, such as midwives [32, 82, 83]. In our descriptive analyses, midwives were identified as comparatively prominent, with an average score of almost 3, indicating a certain level of approval. In previous research, affected women reported that they wished to talk about their experiences of IPV with their midwives but they did not because they thought the midwives could not help them [35]. Strengthening midwives’ skills and strategies to raise the issue of IPV may reduce women’s barriers to talk about their experiences [35]. Investing in training midwives to identify IPV could prove beneficial due to their frequent home visits, as well as their interactions with partners, providing opportunities for IPV detection. Moreover, the oftentimes established trust between women and midwives further enhances the potential for effective intervention. It could also be of advantage to collocate IPV services with health care settings to reduce transportation and time barriers and improve safety and comfort by providing plausible “other reasons” for visiting a facility [82, 83].

### Mode of service provision preferences

Although there is an increasing number of digital offers, women in our sample preferred personal contact. This result is consistent with previous findings [84]. When comparing affected and non-affected women, we found lower preference ratings by women affected by physical and/or sexual IPV compared to non-affected women in the subscale for direct modes. However, there were no significant group differences in their preferences for modes of service provision in total and in their preferences for indirect modes of service provision. The fact that there was no difference in the subscale of indirect modes may stem from the overall lack of popularity of all indirect modes within the entire sample. However, it is conceivable that non-affected women may underestimate the barriers or the overarching circumstances. Consequently, it seems reasonable that women experiencing physical or sexual IPV may prefer indirect modes to direct modes of service provision due to potential feelings of shame. In addition, a heightened fear of their partner discovering their actions may also contribute to this preference. A former study revealed that women found it easier to disclose IPV via a computer than in personal contact [85]. Hence, online tools may be an opportunity to reduce barriers in help-seeking.

### Strengths and limitations

A strength of this study is the large number of participants. By conducting structured interviews via telephone, we reduced participant burden (e.g., no traveling required) and increased anonymity (compared to in person interviews), making it easier to share sensitive experiences [86–88]. The assessment of IPV using the WHO-VAWI allows a valid differentiation of various types of IPV [54]. Further, this is one of very few studies in Germany that quantitatively examine service preferences in the case of IPV based on a theoretical framework [21]. This theoretical framework established a connection between predisposing variables (such as age or duration of residence in Germany), past and present stress exposure (such as IPV), and the preference for various support services [21]. We compared non-affected women and women with experiences of different types of IPV and considered several potential covariates as well as a wide range of services offered by different providers in specific settings and provision modes. By focusing our investigation on women in the postpartum period, we have provided IPV prevalence rates for this special population and deepened our comprehension of their distinct requirements and preferences. Moreover, this has enabled us to incorporate specialized services, such as midwifery care, aimed at addressing the unique needs of women within the postpartum subgroup.

Despite these important contributions, this study also has some limitations. Most of the women in our study sample were born in Germany, had an above-average level of education and income, and lived in Dresden and the surrounding area. Thus, caution should be taken in generalizing our results to other populations. Furthermore, it is important to note that the exclusion of women lacking sufficient proficiency in German or English may have resulted in recent immigrants being underrepresented in our sample.

Regarding our assessment methods, it is possible that the true prevalence rates of IPV are higher than those reported in our study, as it is assumed that there is a dark figure of IPV that goes unreported due to stigma and shame [7], which could have been the case in a telephone interview. Besides that, economic IPV was not separately assessed in this study. To assess women’s preferences with regard to available services within the German support system in Dresden [21], self-generated questionnaires were used. This may reduce the generalisability to other healthcare systems. Besides the wide range of integrated services in our service questionnaire, some services (e. g., police or legal services) were still missing.

Moreover, the study was conducted partly during the COVID-19 pandemic. Compared to many other countries worldwide, the infection protection measures implemented in Germany did not have the direct negative consequences that were partly expected, such as increased IPV rates [89–91]. However, services had to significantly adapt their operations. Counseling services were partially shifted to telephone or digital formats to maintain access to support. Occupancy rates in some shelters had to be reduced, and strict hygiene and distancing measures were enforced. Therefore, it cannot be ruled out that these factors influenced preferences regarding specific support services.

Regarding statistical analyses, not all assumptions were fully met for every analysis. Although additional measures were taken and robust procedures were used whenever possible, it cannot be excluded that insufficient assumptions may have affected the results. Finally, IPV against men and violence against children was not assessed in this study; however, this should not diminish their significance.

### Implications for research

There is a need for studies that investigate the decision-making process itself to better understand how to assist women affected by IPV in their decision to start looking for counseling and treatment services. Thus, our examination so far has been limited to theoretical assessments of utilization or preferences. The subsequent phase entails delineating the sequential stages leading up to the decision to seek assistance or abstain from doing so. This step is crucial to strategically intervene at pivotal points in the help-seeking process. In addition, it is necessary to examine the unmet needs of women which may have led to the lower preference ratings in our study. It is further important to examine the extent to which the severity and duration of IPV as well as being affected by more than one type of IPV influences the preferences.

In addition to whether or not women are affected by IPV, other variables can impact service preferences and need to be assessed. For instance, past and present stress exposure, past and present health, and enabling resources, such as social support and knowledge of existing services or health beliefs, need to be considered. In our study, the awareness about the existence of services was not assessed. As a recently published German study suggests that support services are less known among women affected by IPV compared to those who are not [73], future research should investigate whether the knowledge about services may influence preferences. Subsequent surveys should also ask in detail about women’s preferences regarding additional service settings, such as the police, lawyers, or judiciaries, and add research on the reasons why or why not women may seek help from these services. Moreover, as immigrant women are also at risk of IPV and may face additional difficulties in navigating support systems and accessing help, resulting in distinct preferences for the provision of support services [63], further research is warranted to address the needs of these women as well. Lastly, future studies should investigate why the potential benefits of online interventions [50, 85, 92] were not reflected in women's overall preferences for service provision. To this end, it is important to know under which circumstances women could envision using such services and which concerns may make them reluctant to do so.

### Implications for practice

The results are not only important on a scientific level but also of sociopolitical relevance. First, there is a need for public prevention programs to raise further awareness of IPV and its different forms, its serious impact, and how to support affected women, since acquaintances from the women's social environment were rated as the most preferred types of support. Second, the most preferred psychosocial service were women’s shelters. Women's shelters in Germany are very unevenly distributed and there is a nationwide shortage of more than 14,000 shelter places according to the Council of Europe [93]. It would thus be of paramount importance to initially expand the capacity of women's shelters and concurrently broaden the array of services and provide support not only to women and children who need shelter but also to affected women with other needs, such as counseling, advocacy, or support in legal proceedings. Furthermore, there is a need for better implementation of IPV services in medical settings, as women may access services through healthcare settings [29]. When providing direct or indirect services, professionals should be particularly sensitive to the fact that affected women seem to feel more uncomfortable in direct contact than non-affected women and take measures to reduce potential stigma-related or other fears women might have. Moreover, there is a need for dissemination of knowledge about the availability and potential of online interventions, because they could be of great benefit to affected women.

## Conclusion

This study addressed knowledge gaps related to preferences for counseling and treatment services and the mode of service provision among postpartum women when being exposed to IPV. The results showed that being affected by any type of IPV was associated with lower scores in counseling and treatment service preferences compared to non-affected women. This indicates that there still seem to be too many barriers for many affected women to actually make use of services. Studies examining barriers faced by affected women have identified instrumental barriers such as transportation issues, lack of access to technology, and lengthy waitlists for services. Our findings underscore the importance of addressing these barriers. Further research should investigate which of these potential barriers lead to these lower preferences. Overall, knowledge about the different types of IPV as well as support options should be further disseminated. Efforts should be made to increase awareness of IPV and its impact on mothers and children as well as to improve the availability of service systems such as women’s shelters. IPV screenings need to be implemented in medical settings and health professionals should be trained in detecting IPV. As affected women had a lower preference for a range of services, there is a critical need for health professionals to be competent in providing an effective first-line response through empathetic listening, validating concerns, helping to identify the abuse, and listing options for support and safety [72]. Additionally, there is a need for better implementation of online interventions to make them more popular and available.

## Data Availability

The dataset analysed during the current study is not publicly available due to legal and ethical constraints. Public sharing of participant data was not included in the informed consent of the study. The dataset is available from the corresponding author on reasonable request.

## Appendix 1

**Table:** Rotated factor matrix of principal axes factor analysis (PFA) for the items of counseling and treatment service preferences

Item	Factor loading		
	1	2	3
**Factor 1: Psychosocial services**			
Counseling service for victims of crime	0.642		
Intervention center	0.636		
Psychosocial crisis service	0.584		
Help hotline for violence against women or sexual abuse	0.560		
Life and family counseling center	0.572		
Women's shelter	0.511		
Social pedagogical family assistance	**0.489**		0.338
Telephone counseling	0.492		
Self-help group	0.385		
Outpatient clinic for psychiatry or psychosomatic medicine	**0.357**	0.301	
**Factor 2: Medical services**			
General Practitioner		0.763	
Pediatrician		0.698	
Gynecologist		0.646	
Emergency Room		0.551	
**Factor 3: Midwives**			
Family midwife			0.835
Midwife		0.407	**0.632**

Extraction method: PFA using varimax rotation with fixed number of factors (3) and Kaiser Normalization, three items (“family member, friend, or colleague”, “woman in the same situation” and “religious institutions”) were excluded from analysis

## Appendix 2

**Table:** Rotated Factor Matrix of Principal Axes Factor Analysis (PFA) for the Items of Mode of Service Provision Preferences

Item	Factor loading	
	1	2
**Factor 1: Direct modes**		
Video conference	0.720	
Telephone call	**0.477**	0.366
In Person	0.371	
**Factor 2: Indirect modes**		
App or online platform with guidance from expert		0.753
App or online platform without guidance from expert		**0.7**45
E-Mail		0.675
Chat		0.661

Extraction method: PFA using varimax rotation with Kaiser Normalization

## Appendix 3

**Table:** Spearma﻿n correlations matrix including potential covariates and outcome variables

Variable	1	2	3	4	5	6	7	8	9	10	11	12
1. Maternal age	─											
2. Residence^a^	0.01	─										
3. Income	0.18^**^	0.03	─									
4. Number of children	0.33^**^	0.02	0.05^**^	─								
5. Last 12 months	-0.01	0.00	-0.03	0.07^**^	─							
6. Services^b^	**0.05**^******^	0.01	-0.01	0.03	-0.05	─						
7. Psychosocial^c^	0.03	0.00	-0.00	0.01	-0.02	0.85^**^	─					
8. Medical^c^	0.08^**^	-0.02	-0.02	0.05^**^	-0.08^**^	0.69^**^	0.30^**^	─				
9. Midwives	-0.01	-0.05^**^	0.00	0.02	-0.05	0.60^**^	0.29^**^	0.43^**^	─			
10. Modes^d^	0.03	**-0.04**^******^	0.03	**-0.04**^*****^	-0.02	0.25^**^	0.29^**^	0.09^**^	0.09^**^	─		
11. Direct^e^	0.06^**^	0.03	0.13^**^	-0.04^**^	-0.01	0.30^**^	0.35^**^	0.10^**^	0.09^**^	0.65^**^	─	
12. Indirect^e^	0.00	-0.08^**^	-0.03^*^	-0.03^*^	-0.02	0.14^**^	0.16^**^	0.05^**^	0.06^**^	0.88^**^	0.25^**^	─

Significant correlations between covariates (maternal age, duration of residence in Germany, income, number of children, 12 months prevalence) and outcome variables (services and modes) are printed in bold

^a^Duration of residence in Germany, ^b^Counseling and treatment service preferences, ^c^Services, ^d^Mode of service provision preferences, ^e^Modes

**p* < 0.05*. **p* < 0.01

## Abbreviations

ANCOVA: Analysis of Covariance
APA: American Psychological Association
BCa: Bias-corrected and accelerated
CI: Confidence interval
DFG: Deutsche Forschungsgemeinschaft
INVITE: INtimate partner VIolence Treatment prEferences
IPV: Intimate partner violence
IQR: Interquartile range
LL: Lower limit
M: Mean
MANCOVA: Multivariate analysis of covariance
PFA: Principal axes factor analysis
REDCap: Research electronic data capture
SD: Standard deviation
UL: Upper limit
WHO: World Health Organization
WHO-VAWI: World Health Organization-Violence Against Women Instrument
